# An Automated Approach for Epilepsy Detection Based on Tunable* Q*-Wavelet and Firefly Feature Selection Algorithm

**DOI:** 10.1155/2018/5812872

**Published:** 2018-09-10

**Authors:** Ahmed I. Sharaf, Mohamed Abu El-Soud, Ibrahim M. El-Henawy

**Affiliations:** ^1^Department of Computer Science, Faculty of Computers and Information, El-Mansoura University, Egypt; ^2^Deanship of Scientific Research, Umm Al-Qura University, Mecca, Saudi Arabia; ^3^Department of Computer Science, University College of Umluj, Tabuk University, Saudi Arabia; ^4^Department of Computer Science, Faculty of Computers and Information, El-Zagazig University, Egypt

## Abstract

Detection of epileptic seizures using an electroencephalogram (EEG) signals is a challenging task that requires a high level of skilled neurophysiologists. Therefore, computer-aided detection provides an asset to the neurophysiologist in interpreting the EEG. This paper introduces a novel approach to recognize and classify the epileptic seizure and seizure-free EEG signals automatically by an intelligent computer-aided method. Moreover, the prediction of the preictal phase of the epilepsy is proposed to assist the neurophysiologist in the clinic. The proposed method presents two perspectives for the EEG signal processing to detect and classify the seizures and seizure-free signals. The first perspectives consider the EEG signal as a nonlinear time series. A tunable* Q*-wavelet is applied to decompose the signal into smaller segments called subbands. Then a chaotic, statistical, and power spectrum features sets are extracted from each subband. The second perspectives process the EEG signal as an image; hence the gray-level co-occurrence matrix is determined from the image to obtain the textures of contrast, correlation, energy, and homogeneity. Due to a large number of features obtained, a feature selection algorithm based on firefly optimization was applied. The firefly optimization reduces the original set of features and generates a reduced compact set. A random forest classifier is trained for the classification and prediction of the seizures and seizure-free signals. Afterward, a dataset from the University of Bonn, Germany, is used for benchmarking and evaluation. The proposed approach provided a significant result compared with other recent work regarding accuracy, recall, specificity, F-measure, and Matthew's correlation coefficient.

## 1. Introduction

Epilepsy is a chronic brain disease that affects people of all ages. According to the World Health Organization (WHO), approximately 65 million people suffer from this disorder [1], the majority of whom reside in developing countries and cannot obtain adequate medical treatment. Epilepsy doubles or triples the probability of sudden death when compared with that for healthy people [2]. Moreover, epileptic patients suffer from social stigma and discrimination in their communities. This stigma has a negative impact upon the quality of life of patients and their families. Therefore, the investigation of epilepsy detection techniques and antiepileptic drugs could increase the probability of those coping with this disease to live healthily without social stigmas.

Epilepsy is usually characterized by two or more unprovoked seizures, which affect the ictal person at any time. An elliptic seizure is defined as an excessive electrical discharge in an arbitrary portion of the brain. This rapid discharge causes a disturbance and abnormal behavior in the nervous system. An adequate clinical tool used to recognize epileptic seizures is the EEG signal analysis, as it measures the electrophysiological signals of the brain in real time and measures brain conditions efficiently [3]. However, EEG signal analysis has some limitations in detecting elliptic seizures because of epilepsy behavior such as the following:

1. The occurrence of some seizures is not always because of the epilepsy disorder, as approximately 10% of healthy people may suffer from one seizure in their lifetime. These nonepileptic seizures are similar to epileptic seizures, but they are not related to epilepsy [2]. Hence, the classification of both epileptic and nonepileptic seizures is further significant.
2. Although qualified professional neurologists can visually detect epileptic seizures from an EEG data sheet, it is still considered a time-consuming process.

The diagnostics of epilepsy are usually performed by manual inspections of the EEG signals which not an easy task and requires a highly skilled neurophysiologist. Also, the manual inspection of a long interval recording is a tedious and time-consuming process. Therefore, an intelligent clinical computer-aided design (CAD) tool that analyzes the EEG signal and detects the epileptic seizure is required.

Various case studies have reported the advantages of using automated methods to recognize epileptic seizures from EEG signals. Many techniques are commonly employed for automated EEG analysis and epilepsy detection. Most of these techniques consist of two stages: the first is concerned with feature extraction from the raw EEG signal; the other is dedicated to classifying the features [2]. The feature extraction process is concerned with obtaining significant information from the raw EEG data as well, as it could be implemented in the time, frequency, and time-frequency domains. The time domain and frequency domain are used for signal processing when the EEG is assumed to be a stationary signal. On the other hand, when the EEG signal is considered nonstationary [4, 5], then the time-frequency domain is employed. Case studies demonstrated that the time-frequency domain is more suitable for EEG signal analysis and could obtain significant results [2]. Many algorithms have been proposed for elliptic seizure detection within the time-frequency domain such as empirical mode decomposition (EMD) [6, 7] and wavelet transformation [8–10]. The EMD methods provided a leading trend to detect elliptic seizures from the EEG signal. The EMD has been combined with 2D and 3D phase space representation (PSR) features to identify elliptic seizures. Then, a least-squares support vector machine (LS-SVM) is used to perform the classification process [11]. A combination of different intrinsic mode functions (IMFs) is constructed as a set of features to utilize the classification problem [12]. The EMD has also been used to decompose an EEG signal into a collection of symmetric and band-limited signals. Then, a second-order difference plot (SODP) is applied to obtain an elliptical area. The area under this shape with 95% confidence is used as a selection measure fed to an artificial neural network (ANN) to determine the seizures and seizure-free signals [6]. Although the EMD methods proved their effectiveness, these methods suffer from the mode-mixing problem, which produces intermediate signals and noise. Local Binary Pattern (LBP) based methods represents a different approach of the epilepsy detection. The work presented by [13] suggested a feature extraction based on one dimensional LBP to classify the epileptic seizure, seizure-free, and the healthy classes from the EEG signal. In [14], the researchers have implemented a technique based on the combination of the LBP and the Gabor filter of the EEG signals. Then, the k-nearest neighbor classifier was used for the classification of epileptic seizures and seizure-free signals. The wavelet transformation is usually employed with nonlinear measures to recognize seizures and seizure-free patients from raw EEG signals. An automatic epilepsy detection approach proposed by [15] used the discrete wavelet transformation (DWT) for signal decomposition and generated a feature set using improved correlation-based feature selection (ICFS). Then, the random forest classifier is applied for classification. The DWT has been used with many nonlinear features, and the effectiveness of this approach has been proved [16–23]. Although wavelet transformation is an effective method for EEG signal analysis, this transformation has some limitations [24]. The selection of an appropriate wavelet bias is vital in the time-frequency signal analysis.

A flexible wavelet transformation proposed by [25], namely, tunable* Q*-wavelet transformation (TQWT), controls the transformation of a discrete time signal by an easily tunable variable called the* Q*-factor. The TQWT solved the primary limits of the wavelet filter banks by providing a tunable* Q*-factor that controls the number of the oscillations of the wavelet transformation. Moreover, the TQWT decreased the search space of filter banks by providing three variables only for adjusting. Also, many researchers applied TQWT for physiological signal analysis and proved its effectiveness [21, 22, 26, 27]. However, the after-mentioned methods provided a static set of features (e.g., statistical, nonlinear, and spectral) and did not discuss the adaptive behavior of these features as a dynamical system.

In this paper, an intelligent computer-aided design (CAD) tool that analyses the EEG signal and classifies the epileptic seizure and the seizure-free signal from the input EEG. That provides an asset to the neurophysiologist in interpreting the EEG and reduces the diagnostics time. The proposed method is based on data fusion of a single-channel EEG signal and an image processing approach. In the single-channel EEG signal, the EEG data are processed as a time-frequency time series. The signal is divided into smaller segments of data using tunable Q-wavelet. Some statistical features are extracted from this time series in the time domain and frequency domain. On the other hand, an image processing technique extracts the significant texture from the medical image. Thus, the gray-level co-occurrence matrix is applied to the image, and the contrast, correlation, energy, and homogeneity are extracted. The data fusion approach is used to combine these features of the input EEG signal and construct a large dataset for each patient. Because of a large number of the extracted features, a feature reduction algorithm is needed to reduce the processing time by obtaining a compact subset of features instead of the original one. Moreover, the feature reduction algorithm selects the relevant features, removes redundant features, and discovers the dependency among these features. Therefore, the firefly algorithm is used to find the optimal subset of features. Consequently, bootstraps are obtained by resampling the compact subset to train the random forest classifier. The final decision is obtained by performing a vote for each decision tree of the forest. Hence, the classification of seizure and seizure-free is obtained. A real-world dataset from the University of Bonn is used for benchmarking and validation of the proposed method. A numerical experiment has been implemented, and a comparative study presented a promising efficiency of the proposed system regarding the overall accuracy, sensitivity, and specificity.

The remainder of this manuscript is organized as follows: the preliminaries concepts were introduced in Section 2.  Section 3 introduced the combinational hybrid system of the epilepsy detection. The experiment and discussion were presented in Section 4. Lastly, the paper was concluded in Section 5.

## 2. Preliminary Knowledge

### 2.1. Tunable Q-Wavelet Transformation (TQWT)

The tunability of the* Q*-factor provided a proficient method to adopt the wavelet transformation [25]. The TQWT have three inputs:* Q*-factor denoted by *Q*, which determines the number of oscillations of the wavelet; the number of the oversampling rate, which is denoted by *r* and which determines the number of the overlapping frequency responses; and the number of stages of decomposition, denoted by *J*. For each decomposition stage, the target signal *s*[*n*] with a sample rate of *f*_*s*_ could be represented by low-pass and high-pass subbands with sampling frequencies of *αf*_*s*_ and *βf*_*s*_, respectively, where *α* and *β* are the parameters of signal scaling. The low-pass subband is presented by low-pass filter *H*_0_(*ω*) and low-pass scaling *LPS*(*α*). Similarly, the high-pass subband *ω*_1_ is produced by *H*_1_(*ω*) and *HPS*(*β*). The low-pass and high-pass subband signals are formulated as follows:

(1) Hc0ω=0,απ≤ω≤πθω+β−1πα+β−1,1−βπ≤ω<απ1,ω<1−βπ

(2) H1ω=0,ω<1−βπθαπ−ωα+β−1,1−βπ≤ω<απ1,απ≤ω≤π,

where *θ*(*ω*) could be defined as follows:

(3) $$\begin{array}{c} \theta \left(\;\omega \;\right)=0.5\left(\;1+\cos\left(\;\omega \;\right)\;\right){\left(\;2-\cos\left(\;\omega \;\right)\;\right)}^{0.5},\;\left|\omega \right|\leq \pi \end{array}$$

Both of *r* and *Q* could be represented as filter-bank variables *α* and *β* as follows:

(4) r=β1−α,Q=2−ββ.

### 2.2. Feature Sets

The feature sets used in this research are grouped into four main groups which are statistical, power spectrum, chaotic features, and gray-level co-occurrence matrix (GLCM). The first group contains a set of five features calculated from the time domain of the input signal. This feature set contains mean (*μ*), standard deviation (*STD*), variance (var), Shannon entropy (*H*), and approximate entropy (ApEn). The mathematical formulation of each feature is shown as follows [28–31]:

(5) μx=1N∑i=1Nxi

(6) STDx=1N−1∑i=1Nxi−μx2

(7) varx=1N−1∑i=1Nxi−μ2

(8) HX=∑i=1NPxilog⁡Pxi

(9) ApEnx=ϕmr−ϕm+1r.

The second set of features calculates the power spectrum of the input signal based on the frequency domain analysis. This feature set contains spectral centroid (*SC*), spectral speed (*SS*), spectral flatness (*SF*), spectral slope (*SSI*), and spectral entropy (*PSE*), where *Y*(*q*) denotes the for the discrete Fourier transformation of the input signal *f*(*n*). The mathematical formulation of each feature is shown as follows [32]:

(10) SC=∑q=0M−1qYq∑q=0M−1Yq

(11) SS=∑q=0M−1q−SC2Yq∑q=0M−1Yq

(12) SF=∏q=0M−1Yq1/M1/M∑q=0M−1Yq

(13) SSI=M∑q=0M−1fmYq−∑q=0M−1fm·∑q=0M−1YqM∑q=0M−1fm2−∑q=0M−1Yq2

(14) PSE=−∑i=1nYq∑i=1nYqln⁡Yq∑i=1nYq

The third set of features contains chaotic measures to obtain the dynamic behavior of the EEG signal. This set includes Higuchi's fractal dimension (*HFD*), Hurst exponent (*H*_*r*_), and Katz fractal exponent (*KATZ*). These features are formulated as follows [33–36]:

(15) HFD=ln⁡kLk

(16) ERnSn=cnHr

(17) KATZ=log⁡nlog⁡n+log⁡d/L

The final set of features consists of statistical measures of an image represented as matrices called gray-level co-occurrence matrix (GLCM) where *C*(*i*, *j*) represents an entry in co-occurrence matrix and *i*, *j* = 0,1, 2,…*L* − 1, where *L* is the number of gray levels in the image. Those matrices represent the spatial dependencies between the gray levels of image reflecting the structure of the underlying texture. After the normalization of these matrices, the contrast, correlation, energy, and homogeneity are computed as follows:

(18) Energy=∑i=0L−1 ∑j=0L−1Ci,j2

(19) Contrast=∑i=0L−1 ∑j=0L−1i−j2Ci,j

(20) Correlation=∑i=0L−1 ∑j=0L−1i−μij−μjCi,jσiσj

(21) Local  homogeneity=∑i=0L−1 ∑j=0L−111+i−j2Ci,j

### 2.3. Firefly Optimization Algorithm

The firefly algorithm is a swarm based stochastic search technique [37]. The firefly optimization algorithm consists of a set of members called fireflies; each firefly represents a candidate solution. The most attractive firefly is considered to be the leader firefly that leads the other candidates to the best region. The attractiveness is calculated based on the light intensity which is usually determined by the objective fitness function. The attractiveness between two fireflies *X*_*i*_ and *X*_*j*_ is determined as follows:

(22) βrij=β0e−γrij2

(23) rij=∑d=1Dxid−xjd2

where *D* denotes the problem dimension such that *D* = {1,2,…, *d*}, *r*_*ij*_ denotes the distance between *X*_*i*_ and *X*_*j*_. Parameter *β*_0_ denotes the initial attractiveness at *r* = 0 and *γ* denotes the light absorption factor such that *γ* ∈ [0,1]. Each firefly *X*_*i*_ is compared with the other fireflies *X*_*j*_ where *j* ∈ {1,2,…*N*} such that *i* ≠ *j* and *N* denotes the count of the fireflies. If firefly *X*_*i*_ is better (brighter) than *X*_*j*_, then firefly *X*_*j*_ moves towards *X*_*i*_ with a step movement formulated as follows:

(24) Xidt+1=Xidt+β0eij−γrij2Xjdt−Xidt+αϵi

where *ϵ*_*i*_ represents uniform a randomly distributed variable such that *ϵ*_*i*_ ∈ [−0.5,0.5] and *α* denotes the movement step such that *α* ∈ [0,1].

## 3. The Combinational Hybrid System of Epilepsy Detection from EEG Signal

**Figure 1:**
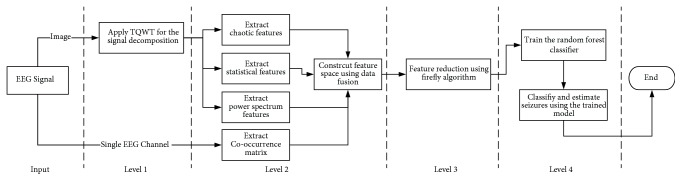
Block diagram of the combinational hybrid system of epilepsy detection from EEG signal with 4 levels of processing.

In this research, a hybrid system was proposed to detect both seizures and seizure-free conditions from a raw EEG signal. Although some investigations focused on the feature extraction level, the proposed system was established based on four main levels. This system combined the data fusion approach with firefly optimization and random forest. The *TQWT* was applied for EEG signal decomposition; then the features were constructed using a data fusion technique. Due to the large number of features obtained for each subband (*featuresCount* × *J* × *Q*), a feature reduction was applied to reduce the features and to obtain a compact set of features instead of the original one. The obtained compact set of features was fed to a random forest algorithm to obtain the classification rules and hence used for training. After training, the classifier should be able to classify and estimate the preictal phase. The proposed system was divided into the following four levels of processing and then described in detail as shown in Figure 1.

- EEG decomposition using TQWT
- Feature extraction using data fusion based on single-channel EEG signal and co-occurrence matrix
- Feature reduction using firefly optimization algorithm
- Training of random forest classifier to detect the seizures and seizure-free EEGs

### 3.1. EEG Decomposition Using TQWT

**Figure 2:**
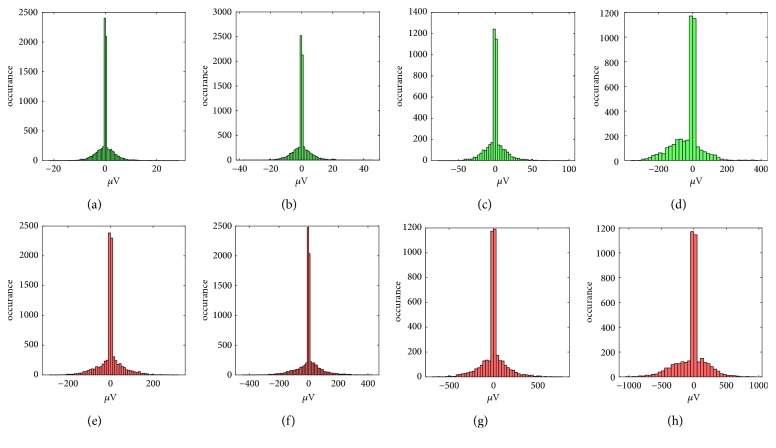
Illustration of seizure-free and epileptic seizures of subbands obtained from TQWT with Q = 1, r = 3, J = 3. Figures (a), (b), (c), and (d) represent the first, second, third, and fourth subbands obtained from seizure-free signals. Figures (e), (f), (g), and (h) represent the first, second, third, and fourth subbands obtained from the epileptic seizures.

The preprocessing level applies the *TQWT* decomposition to the input EEG signal. The *TQWT* converted the continuous EEG signal to discrete potions of data that could be handled more effectively. This wavelet transformation is used because of its effectiveness in signal decomposition and its tunability. The obtained subbands using the *TQWT* provided a significant difference between the seizure-free and the epileptic seizure of the EEG signals as shown in Figure 2. The subfigures denoted by (a), (b), (c), and (d) visualize the histogram of the first, second, third, and fourth subbands of the seizure-free class. The remaining subfigures denoted by (e), (f), (g), and (h) represent the histogram of the epileptic seizure class for the same subbands. The values of the extracted subbands of the second class are about ten times stronger than the first class that prove the efficiency of this decomposition.

### 3.2. Feature Extraction Using Data Fusion Based on a Single-Channel EEG and a Co-Occurrence Matrix

In the first perspective, the EEG signal was described as a nonstationary time series. After the decomposition of the EEG signal using the TQWT, a feature extraction process was performed to obtain significant characteristics from each TQWT subbands. The extracted features were categorized into three main groups. The first group determines the statistical characteristics in the time domain. The mean, standard deviation, variance, Shannon entropy, and approximate entropy were calculated in the first group as formulated from (5) to (9). This group indicates some statistical information obtained from the time domain of TQWT subband. The second group of features determines a power spectrum analysis of obtained subbands. The discrete Fourier transformations (DFT) were applied to convert these subbands into a frequency domain. Then the power spectrum features were extracted. The second group consists of the spectral centroid, spectral speed, spectral flatness, spectral slope, and spectral entropy as formulated from (10) to (14). The power spectrum analysis represents an effective method to study the frequency behavior of the signal. The last group of features performs a chaotic analysis of each subband. Because of the nonlinearity of the EEG signals, a nonlinear analysis is required. One of the best analyses used for this issue is the chaotic analysis. In this analysis, the Higuchi fractal dimension (HFD), Hurst exponent, and Katz fractal exponent were computed for each subband as formulated from (15) to (18).

In the second perspective, the input EEG signal was converted to a gray image. Then a co-occurrence matrix was computed to obtain the gray levels of the image. Then the textures of contrast, correlation, energy, and homogeneity were calculated from this matrix to represent the statistical measures of the image as formulated from (19) to (21). Finally, after computing the feature space, a data fusion was applied to merge all of these features and create a single dataset.

### 3.3. Feature Reduction Using Firefly Optimization Algorithm

In this section, a feature reduction algorithm based on the firefly algorithm is proposed [37, 38]. This algorithm implements a chaotic movement, simulated annealing (SA) to produce efficient offspring candidates, and memory awareness of the best and worst solutions to improve the search diversity and prevent local optima. The firefly population is randomly initialized using a chaotic logistic map to ensure the diversely of the candidates and the randomness of each firefly. Afterwards, the fitness function is computed for each candidate and identifies both the best and worst solutions as *g*_*best*_ and *g*_*wosrt*_, respectively. For each iteration, an alternative candidate is declared as *S*_*best*_ with a competitive fitness and located in a different region. Both of the best and the alternative candidate sets are used to lead weak solutions to reach the optimal region and prevent local optima problem. The mean of the leader firefly and the alternative one is enhanced using SA algorithm to obtain a better solution *g*_*best*_′. The improved local and global solutions are used to guide the low lightness fireflies to move towards that stronger lightness. The algorithm is repeated until the maximum number of iterations is reached, or a termination criterion is achieved. The behavior of the proposed algorithm is determined by some properties, namely, the objective function, the attractiveness movement step, and population diversity. The proposed algorithm is demonstrated in Algorithm 1.

*The Objective Function.* This function is used to evaluate each candidate in the algorithm and defined as follows:

(25) $$\begin{array}{c} f\left(\;x\;\right)=w_{1}\times accuracy\left(\;x\;\right)+\frac{w_{2}}{\text{number\_of\_features}} \end{array}$$

where *w*_1_ and *w*_2_ represent the weights of the classification accuracy and the number of the selected features, respectively. The values of *w*_1_ and *w*_2_ are set to 0.9 and 0.1, respectively, as a recommended by [38].

*The Attractiveness Movement Step.* The proposed algorithm used a chaotic logistic map to initialize the firefly population and hence increases the diversity and avoid local optima. After obtaining the global best solution *g*_*best*_, an alternative leader firefly *S*_*best*_ is declared with a competitive fitness but located in a different region. Since both leaders are more likely to discover distinctive search regions, this strategy reduces the probability of being trapped in the local optima. In addition, the optimal offspring of the mean positions of the two leaders and the neighboring brighter candidates are used to lead the search process and guide the solutions with lower light intensity to move towards the optimal region.

(26) xi=xi+β0Ckxj′−xi+Ckεgbest′−xi+α′×signrand−0.5

(27) xj′=xj+σ1

(28) gbest′=meangbest+Sbest+σ2

where *x*_*j*_′ denotes the offspring candidate with a brighter neighboring solution, *x*_*j*_ is defined by the SA as shown in (26), and *g*_*best*_′ represents the fitter offspring solutions of the mean of the leader firefly and the alternative one as formulated in (27). It worth mentioning that the values of *σ*_1_ and *σ*_2_ are two random variables set using the Gaussian distribution. The movement step of the firefly is determined as shown in (28), where *C*_*K*_ represents the chaotic map variable in the movement step and *ε* denotes the randomized vector defined in the traditional firefly algorithm. Parameter *α*′ denotes an adaptive step initialized to 0.5 to control the diversity of the search process. 

*Offspring Generation Using SA.* The proposed algorithm used SA for generating better candidates to enhance the search process as much as possible as shown in Algorithm 2. The SA accepts both of the best solution *g*_*best*_ and the alternative solution *S*_*best*_ as main inputs, then the traditional SA is applied. The better solution generated is accepted by default according to the SA heuristics. On the other hand, the weaker solution should be accepted with specific probability as shown in (29), where Δ*f* denotes the difference of the fitness (energy) between to candidates and *T*_*c*_ denotes the current temperature. A simple linear cooling mechanism is used to control the value of the temperature.

(29) $$\begin{array}{c} p_{x_{j}}=\exp\left(\;\frac{\Delta f}{T_{c}}\;\right) \end{array}$$

*Population Diversity.* In each iteration, the worst solution is detected after the ranking process as shown in Algorithm 1. The remaining solutions are guided by the average position obtained from (30) where *σ* denotes a random value obtained by Gaussian map.

(30) $$\begin{array}{c} x_{j}^{worst}=\frac{g_{best}+S_{best}}{2}+\sigma \left(\;x_{j}^{worst}\frac{g_{best}+S_{best}}{2}\;\right) \end{array}$$

### 3.4. Classification and Learning

The random forest (RF) is a successful ensemble approach used in supervised machine learning to solve classification or regression problems [39]. It consists of a collection of decision trees that could act as a single classifier with multiple classification methods or a method that has several variables. Several subsets of the training data are supplied to each tree to achieve the most stable tree classification that results in a generalized experience of the classifier. The original dataset is divided into two parts. The first part is used to train each tree by bootstrapping technique. The other part is used to evaluate the accuracy of the classification. Each tree is allowed to reach the maximum depth without tree pruning to obtain a high variance classifier. The splitting process remains until only one instance of a single class is dropped from any leaf node or a predefined termination condition is achieved. When the forest is established, the number of subsets remained as a constant. The obtained route of traversal from the root node to the leaf node is applied to the new instances or the unlabeled instance for classification. The final decision for classifying a new instance is provided by determining each class that has the most votes from every decision tree. The random forest performs slightly better when compared with other classifiers such as discriminant analysis, SVM, and artificial neural network (ANN) [15, 39].

### 3.5. Performance Evaluation

Various performance formulas were used to evaluate the effectiveness of a classifier. The sensitivity (SEN) or recall, specificity (SPEC), accuracy (ACC), F-measure, Matthew's correlation coefficient (MCC), and receiver operating characteristics (ROC) are used to evaluate the efficiency of the random forest classifier [40–42]. These parameters are defined as follows:

(31) SEN=TPTP+FN×100

(32) SPEC=TNTN+FP×100

(33) ACC=TP+TNTP+TN+FP+FN×100

(34) F−measure=2TP2TP+FP+FN×100

(35) MCC=TP×TN−FP×FNTP+FNTP+FPTN+FNTN+FP×100

*TP* and *TN* represent the total number of an epileptic seizure and seizure-free signals classified correctly, respectively. Similarly, *FP* and *FN* represent the total number of epileptic seizures and seizure-free signals classified incorrectly, respectively. Cross-validation has also been used to ensure the classifier reliability and effectiveness. The original dataset is split into *k* folds (subsets) for both training and testing. In this strategy, *k* − 1 folds were selected randomly to train the classifier, and the remaining folds are used to testing. The overall performance is calculated as the average of each fold. In this work, the experiment is repeated 10 times with tenfold cross-validation.

## 4. Results and Discussion

The benchmark dataset used in this investigation was acquired by the University of Bonn [28]. The dataset contains three different categories, i.e., preictal, healthy, and ictal recorded using a single channel for a 23.6 s duration. Both normal and preictal conditions were collected from 200 case studies and 100 for the ictal state. The normal condition is acquired from five healthy volunteers using the international 10–20 system standard with each volunteer in a relaxed-awake state with eyes open and closed (100 cases per each set) [28]. The ictal data were collected from five patients during their epileptic seizures. The preictal represents the EEG data collected from the same five patients with no seizures. It is worth mentioning that all EEG signals were acquired using a 128 channel amplifier with sampling rate equal 173.61 Hz [28]. Finally, a bandpass filter with 0.5340 Hz ~12 dB/octave was applied as a filter.

**Figure 3:**
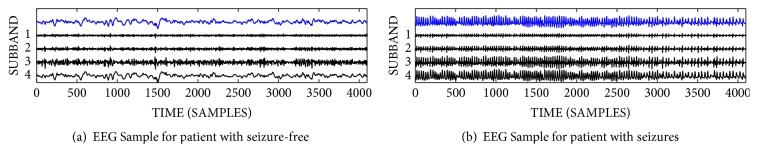
TQWT decomposition of seizure-free and seizure EEG signals with *Q* = 1, *r* = 3, *j* = 3.

The proposed approach for automatic detection of epileptic seizures and seizure-free patients was implemented using MATLAB software. A TQWT comparison between the seizure-free and the epileptic seizures patients are shown in Figure 3 with *Q* = 1, *r* = 3, and *J* = 3. It can be observed that both of the amplitudes and the frequency of the epileptic seizure are much higher than the healthy one. Moreover, the oscillatory behavior of the epileptic patient is higher than the healthy one. The value of the parameter *r* is set to three, to prevent any excessive ringing of the wavelet as suggested by [22]. MATLAB for the TQWT toolbox is available for public access at http://eeweb.poly.edu/iselesni/TQWT/.

**Figure 4:**
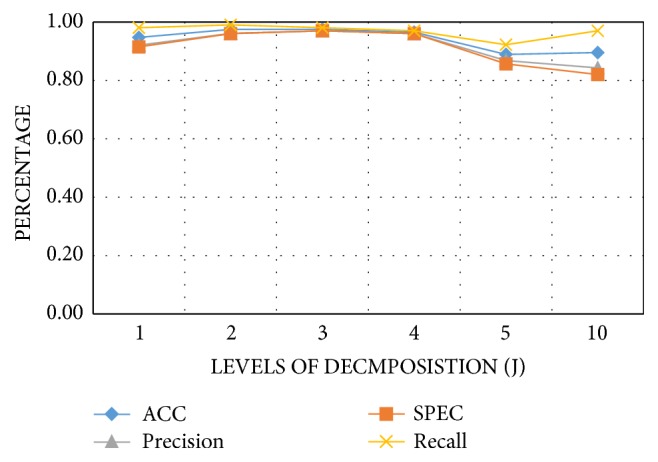
Performance measures of the proposed approach with varied levels of decomposition.

**Figure 5:**
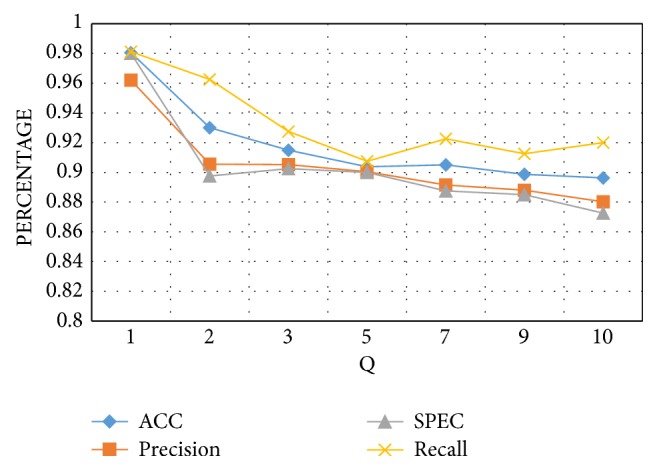
Performance measures of the proposed approach with varied levels of decomposition.

After the construction of the dataset, the feature reduction was applied using the firefly algorithm to remove the redundant and irrelevant features. The number of the data segments was determined by the parameters *Q*, *r*, and *J*. By tuning these parameters, the number of the data segments was varied, and thus the training process was adopted. The trial and error approach was used to set the value of these parameters. The experiment was implemented on various values of J to obtain the best level of decomposition. The performance measures were calculated for each level of decomposition. As shown in Figure 4, the best value of the variable *J* was from two to three. Moreover, the best value of the parameter *Q* was found to be one as shown in Figure 5. All the variables remained constant while changing the* Q*-factor to obtain the best value.

**Figure 6:**
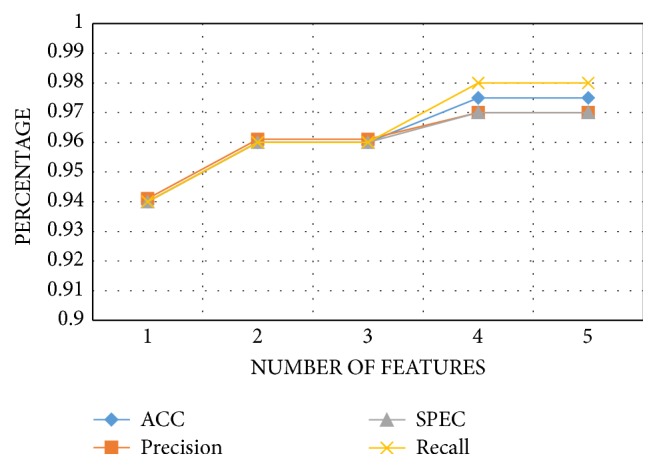
Performance measures of the original and compact features set.

Then, the firefly algorithm with a population size of 20 fireflies, mutation probability of 0.01, and light absorption equal to 0.1 was applied to reduce the feature set. The result of the compact set of features is obtained after 100 iterations and shown in Figure 6 are the number of features obtained by the firefly algorithm and their corresponding accuracy, precision, specificity, and the recall. In the last step, the feature set is fed to a random forest classifier to obtain the seizure-free and seizure conditions. The random forest classifier obtained 98% accuracy, 97% precision, 97% specificity, 98% recall, 98% F-measure, and 95% MCC at the third level of decomposition, and the* Q*-factor equals 1.

**Figure 7:**
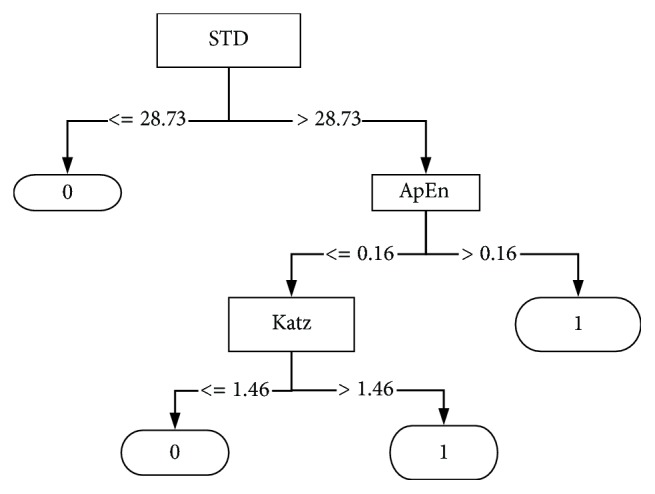
The classification rules obtained from the proposed system to classify the seizure-free (0) and the epileptic seizure (1) signals.

The firefly algorithm reduced the search space into three features. These features could replace the original dataset which minimizes the processing time. The compact set of feature contains the *STD*, *ApEn*, and the *KATZ*; the classification rules are based on these features. The classification rules obtained by the proposed system are shown in Figure 7, where the decision tree consists of 4 leaves and 7 nodes, and the final decision represents the seizure-free and the epileptic seizure denoted by 0,1, respectively.

**Figure 8:**
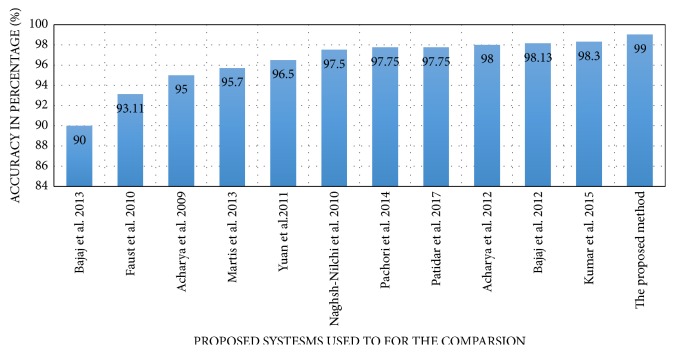
The total accuracy (%) of various methods employed to detect the disorder of the epilepsy.

A comparative study of the proposed hybrid epilepsy detection approach and other existing classification systems has been performed in terms of the total accuracy. A novel method based on the EMDs was proposed to detect the epileptic seizures of epilepsy. This method used the Hilbert transformation of IMFs obtained by EMD process that provided an analytic signal representation of IMFs [12]. The classification rules obtained by this method achieved an accuracy of 90%. The usage of frequency domain features and Burg's method obtained 93.11% accuracy with SVM classifier [43]. Nonlinear features have been used with a Gaussian mixture model classifier and achieved 95% accuracy [44]. A decision tree classifier is used with energy, fractal dimension, and sample entropy and provided 95.7% [45]. A combination of ApEn and the Hurst exponent has been used to detect the diagnostics of epilepsy and produced 96.5% accuracy with SVM classifier and ANN [46]. In [47], an eigensystem based method was proposed cooperated with Multiple Layer Perceptron to classify the epileptic seizures, healthy, and the seizure-free. This approach provided an average accuracy of 97.5%. The EMD methods for epilepsy detection achieved 97.75% accuracy [6]. The Kraskov entropy is also combined with the SVM classifier and provided 97.75% [22]. An automated diagnostics system based on a set of entropies and fuzzy Sugeno classifier (FSC) achieved accuracy up to 98% [19]. The work presented by [48] developed a method for the epilepsy detection using the EMD. The generated IMFs using the EMD were represented as a set of amplitude and frequency modulated (AM–FM) signals. The two bandwidths, namely, amplitude modulation bandwidth and frequency modulation bandwidth, calculated from the analytic IMFs, have been fed to LS-SVM for classifying seizure and nonseizure EEG signals. This method achieved 98.18% average accuracy. The LBP-based methods have been combined with Gabor filter for texture extraction from the EEG signal. A k-nearest neighbor classifier was applied and obtained an accuracy of 98.3% [14]. The proposed method confirmed its superiority in the total accuracy compared to the other systems. The results, which prove the superiority of the proposed method compared to the other existed systems, is demonstrated in Figure 8.

Once the system detects the preictal phase, the clinic receives a notification about that patient. The main advantage of the hybrid system from the clinical point of view could be summarized as follows:

- Classification and detecting of the epileptic seizures and seizure-free signals from the EEG signal automatically
- The detection of the preictal phase from studying the healthy and ictal phase of each patient
- The detection of the preictal phase that provided the ability to send warning alert to the physicians to prepare the medical assessment for the patient
- The proposed method being robust and reliable as its performance was benchmarked using 10-fold cross-validation
- The dynamic behavior obtained from the firefly algorithm which made the system adaptive to many features according to each case study.
- A few sets of parameters required to analyze the EEG signal typical three variables (*Q*, *r*, *J*).

The limitations of this research were summarized as follows:

- The limited number of the studied subjects (typically 100 per class)
- The diagnosis process that may be reduced because of depending on some additional software prerequisites.

## 5. Conclusion

In this paper, an automated intelligent CAD tool has been proposed to classify and detect epileptic seizures and seizure-free EEG signals. This method provided an EEG signal analysis using a hybrid data fusion method. The data fusion method combined the collected features from two different perspectives. In the first perspective, the EEG signal was considered as an image. Then, the image was converted to a gray image and the GLCM was obtained to extract the textures of the image such as contrast, correlation, power, and homogeneity. In the second perspective, the EEG signal was divided into smaller segments using the TQWT to extract the time and frequency features. A set of statistical, nonlinear (chaotic), and power spectrum features were obtained from each segment. After the dataset was constructed, a feature reduction algorithm based on firefly optimization was used to reduce the irrelevant features and remove redundancy. Then, an RF was trained to classify and predict the epileptic seizures and seizure-free EEG signals from the dataset. The experimental results showed that the proposed method achieved a satisfactory degree of 99% accuracy, 97% precision, 97% specificity, 98% recall, 98% F-measure, and 95% MCC at the third level of decomposition.

## Figures and Tables

**Algorithm 1:**
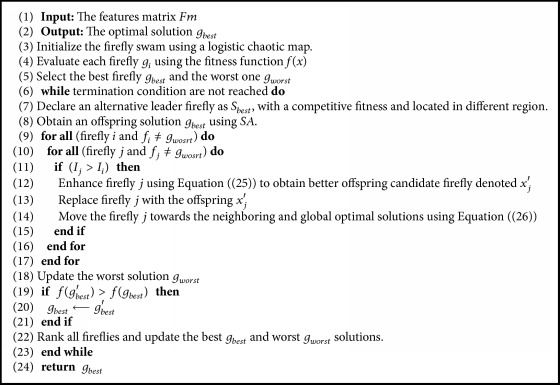
The pseudocode of the proposed feature reduction algorithm based on firefly optimization and SA.

**Algorithm 2:**
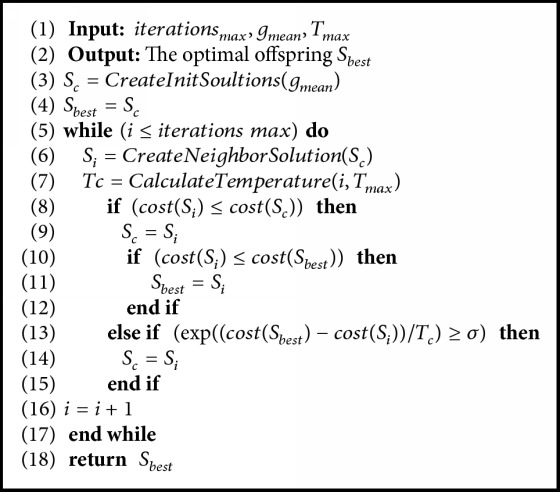
The pseudocode of the generation of offspring solutions using SA.

## Data Availability

The dataset used to support the findings of this research was included in the article and can be found as a citation to “R.G. Andrzejak, K. Lehnertz, F. Mormann, C. Rieke, P. David, C.E. Elger, Indications of Nonlinear Deterministic and Finite-Dimensional Structures in Time Series of Brain Electrical Activity: Dependence on Recording Region and Brain State, Physical Review E, 64 (2001) 061907”.
